# Chronic Kidney Disease Screening Methods and Its Implication for Malaysia: An in Depth Review

**DOI:** 10.5539/gjhs.v7n4p96

**Published:** 2014-12-31

**Authors:** Yasmin Almualm, Hasniza Zaman Huri

**Affiliations:** 1 Clinical Investigation Centre, Faculty of Medicine, University of Malaya, Kuala Lumpur, Malaysia; 2 Department of Community Health, Faculty of Medicine, National University of Malaysia, Malaysia; 3 Department of Pharmacy, Faculty of Medicine, University of Malaya, Kuala Lumpur, Malaysia

**Keywords:** albuminuria, Chronic Kidney Disease (CKD), Chronic Kidney Disease Epidemiology Collaboration (CKD-EPI), creatinine, Cystatin C, Glomerular Filtration Rate (GFR)

## Abstract

Chronic Kidney Disease has become a public health problem, imposing heath, social and human cost on societies worldwide. Chronic Kidney Disease remains asymptomatic till late stage when intervention cannot stop the progression of the disease. Therefore, there is an urgent need to detect the disease early. Despite the high prevalence of Chronic Kidney Disease in Malaysia, screening is still lacking behind. This review discusses the strengths and limitations of current screening methods for Chronic Kidney Disease from a Malaysian point of view. Diabetic Kidney Disease was chosen as focal point as Diabetes is the leading cause of Chronic Kidney Disease in Malaysia. Screening for Chronic Kidney Disease in Malaysia includes a urine test for albuminuria and a blood test for serum creatinine. Recent literature indicates that albuminuria is not always present in Diabetic Kidney Disease patients and serum creatinine is only raised after substantial kidney damage has occurred. Recently, cystatin C was proposed as a potential marker for kidney disease but this has not been studied thoroughly in Malaysia. Glomerular Filtration Rate is the best method for measuring kidney function and is widely estimated using the Modification of Diet for Renal Disease equation. Another equation, the Chronic Kidney Disease Epidemiology Collaboration Creatinine equation was introduced in 2009. The new equation retained the precision and accuracy of the Modification of Diet for Renal Disease equation at GFR < 60ml/min/1.73m^2^, showed less bias and improved precision at GFR>60ml/min/1.73m^2^. In Asian countries, adding an ethnic coefficient to the equation enhanced its performance. In Malaysia, a multi-ethnic Asian population, the Chronic Kidney Disease Epidemiology Collaboration equation should be validated and the Glomerular Filtration Rate should be reported whenever serum creatinine is ordered. Reporting estimated Glomerular Filtration Rate will help diagnose patients who would have been otherwise missed if only albuminuria and serum creatinine are measured.

## 1. Introduction

The higher incidence of non-communicable diseases, notably diabetes and hypertension, has lead to an increase in the number of CKD patients. In the US, 25.8 million people have Diabetes (8.3% of the US population) (*National diabetes fact sheet: National estimates and general information on diabetes and prediabetes in the united states*, 2011). Diabetes is the leading cause of Chronic Kidney Disease (CKD) in developed countries. It is estimated that 15-20% of type 1 diabetes patients and 30-40% of type 2 diabetes patients will develop end stage renal disease (Benz & Amann, 2011). In Malaysia, Diabetes is the leading cause of CKD contributing to 58% of all new patients requiring dialysis in 2012 (Lim, 2013). Although CKD remains asymptomatic till late stage, the progressive renal function decline during the course of diabetic nephropathy can be detected early when renal function is still normal or elevated (Perkins et al., 2007). Early diagnosis is important for kidney disease patients as it is a source of considerable morbidity and mortality. The most widely used definition of CKD is by the National Kidney Foundation’s Kidney Disease Outcome Quality Initiative stating Glomerular Filtration Rate (GFR) <60ml/min/1.73m^2^ that is present for 3months or more; or evidence of kidney damage for 3 months or more with or without decreased GFR as evidenced by any of the following: Microalbuminuria, Macroalbuminuria, Proteinuria, Glomerular Haematuria, Pathological abnormalities, Anatomical abnormalities (K/doqi clinical practice guidelines for chronic kidney disease:Evaluation, classification, and stratification. Am j kidney dis, 2002, 39, S1-266.). Screening for kidney function includes Glomerular Filtration Rate (GFR) and Albuminuria (Lamb, 2011; A. S. Levey et al., 2003; Stevens & Levey, 2009). In the US and many parts of Europe the GFR is automatically reported when serum creatinine is ordered, this however is not the case in Malaysia. Screening for CKD in Malaysia includes a urine test for albuminuria and a blood test for serum creatinine. The aim of this review is to discuss the strengths and limitation of CKD screening methods and its application in Malaysia, a multiethnic Asian population.

## 2. Methodology

### 2.1 Design and Selection Methods

This is a review of the literature on current CKD screening practices. A systematic search of the literature was carried out and data were retrieved from Google Scholar, Pubmed Central, Scopus and Science Direct. The keywords used were Albuminuria, Chronic Kidney Disease (CKD), Chronic Kidney Disease Epidemiology Collaboration (CKD-EPI), Creatinine, Cystatin C, Glomerular Filtration Rate (GFR). The Search was limited to studies published in the English language for the period between 2000 and 2014. The search included cross-sectional and longitudinal studies carried out among the general population and CKD patients with or without diabetes. The search identified a total of 2040 studies of which 38 studies were selected by the Authors as relevant to the subject under investigation and met the preset inclusion criteria. All 38 studies were carried out in Asian and western countries.

### 2.2 Selection Criteria

#### 2.2.1 Albuminurea

The search for albuminuria identified a total of 866 articles. The selection criteria are:

(1) Cross-sectional studies among diabetic patients who underwent screening for kidney function. Only studies that have reported the number of diabetic patients with GFR <60ml/min/1.73m^2^ and normal albuminuria were selected. Six articles met the criteria.
(2) Longitudinal studies carried out among diabetic patients with and without albuminuria. Studies that reported the course of albuminuria at the end of the study period were selected. Six studies met the criteria.

#### 2.2.2 GFR

The search for GFR equation identified 216 articles. The selection criteria are:

(1) Longitudinal studies among patients with and without diabetes in which the GFR was estimated using the MDRD equation and the CKD-EPI equation. Studies that compared both equations in terms of risk stratification and CKD prevalence were selected. Five articles met the criteria.
(2) Cross sectional studies among patients with a wide range of GFR who underwent GFR estimation using the MDRD equation and the CKD-EPI equation. Studies that compared the performance of both equations in terms of bias and accuracy were selected. Six articles met the criteria.

#### 2.2.3 Cystatin C

The search for cystatin C identified 958 articles including patients with and without CKD. The selection criteria are cross-sectional studies among CKD patients in which serum cystatin C was measured. Studies that compared serum cystatin C with serum creatinine for detecting renal disease were included. Thirteen articles met the criteria.

### 2.3 Data Extraction

The following variables were extracted from the selected studies, study design, population charecteristics, number of participants, number of years of follow up, estimated and measured GFR (when applicable), percentage of patients with normal albuminuria, percentage of patients with microalbuminuria, prevalence of CKD and number of CKD patients reclassified using the CKD-EPI equation.

## 3. CKD Screening in Malaysia

The prevalence of diabetes and its risk factors continue to rise at an alarming rate. From 1996 to 2006 Malaysia witnessed a 250% increase in the prevalence of Obesity and 88% increase in prevalence of diabetes (*National strategic plan for non-communicable disease*, 2010). Recently the prevalence of diabetes among adults age 18 years and above in Malaysia is reported at 15.2% (*National health and morbidity survey report*, 2011). Uncontrolled diabetes can lead to complications. CKD is one of the long term complications of diabetes. In Malaysia, diabetes is the leading cause of CKD contributing to 58% of all new patients requiring dialysis in 2012(YN Lim, 2013). This has considerable public health implication considering the morbidity and high cost associated with the disease. The prevalence of CKD in Malaysia is currently reported at 9.07% (Hooi et al., 2013). It is recommended that screening for CKD is initiated at the time of diagnosis of type 2 diabetes and 5 years after the diagnosis of type 1diabetes. Screening includes assessment of proteinuria, heamaturia and renal Function (*Clinical practice guidlines: Management of chronic kidney disease in adults*, 2011). Screening is carried out annually for diabetic and hypertension patients. Screening for CKD is also recommended for patients at high risk of CKD. Screening for albuminuria is carried out using urine dipstick. This test is cheap, convenient and widely available. In diabetic patients, urine albumin: creatinine ratio (ACR) in an early morning urine sample should be performed annually if urine dipstick is negative. Microalbuminuria is defined as ACR 2.5-30 mg/mmol for males and 3.5-30 mg/mmol for females. Due to non renal factors affecting albumin level in urine, repeat tests are carried out within three to six months. When 2 out of 3 tests are positive then patient is diagnosed with diabetic nephropathy. Renal function will be assessed by estimated GFR using the Modification of Diet in Renal Disease (MDRD) equation. Staging of CKD is based on KDIGO 2002 (National kidney foundation. Kdoqi clinical practice guidelines for chronic kidney disease: Evaluation, classification and stratification. [KDIGO], 2002). Other tests such as renal imaging or biopsy are carried out when deemed necessary. Currently, it is not a common practice for laboratories in Malaysia to report an estimated GFR when renal profile or serum creatinine is ordered by physicians. In practice, serum creatinine is used by clinicians to determine the presence or absence of CKD despite its known limitation (Chia Yook Chin, 2012). A recent study carried out at primary care setting in Malaysia showed that using serum creatinine instead of estimated GFR to detect CKD fail to detect a third (35.5%) of patients with renal impairment who had seemingly normal serum creatinine (Chia Yook Chin, 2012). This is similar findings to another study carried out in Spain that found that estimated GFR identified an additional 13.4% of diabetic patients with renal impairment who would have been missed if only serum creatinine was measured(Coll-de-Tuero et al., 2012). The following sections will discuss albuminuria and GFR as two principle methods for CKD screening. In addition, cystatin C will be discussed as a new potential marker for CKD screening.

## 4. CKD Screening Methods: Strength and Limitation

### 4.1 Albuminuria

The clinical picture of diabetic kidney disease has been characterized by the presence of albuminuria prior to the decrease in GFR. Screening for albuminuria is cheap, convenient and widely available. Microalbuminuria refers to the increase passage of albumin through the glomeruli. The mechanism for the development of microalbuminuria is complex and involves changes in glomerular pressure (or filtration) in addition to changes to glomeruli structure (Satchell & Tooke, 2008). Screening for albuminuria is one of the primary methods to detect kidney disease (Lamb, 2011; Perkins et al., 2007; Stevens & Levey, 2009; Wang, Li, Gong, & Lou, 2013). It should be carried out at least once a year for diabetic patients. Albumin excretion can increase transiently due to non-renal factors as well, such as urinary tract infection, fever, exercise, congestive heart failure and hyperglycemia (Stevens & Levey, 2009). Due to this variation, microalbuminuria is confirmed if at least 2 of 3 tests (performed within minimum of 3 months) are positive. Microalbuminuria is defined by an albumin excretion (AER) between 20-200 µ/min (30-300 mg/24 hours), this amount cannot be detected by urine dipstick. Proteinuria, also known as macroalbuminuria is defined as AER exceeding 200 µ/min (300 mg/24 hours). Macroalbuminuria is associated with decline in GFR and rise in Blood pressure(Chadban S, Mangos G, & Guideline for Diagnosis, 2009). The GFR in people with type 2 diabetes typically begins to decline in the late microalbuminuric stage and, without intervention declines at an average rate of 8-12 ml/min/1.73m^2^/year (Biesenbach, Janko, & Zazgornik, 1994). However, the classical picture of albuminuria prior to declining GFR has been challenged recently. Growing evidence suggests that not all diabetic patients will present with the typical albuminuria findings and that CKD may be accompanied, rather than preceded by microalbuminuria, or it may even develop in those with albuminuria levels that revert to normal (Halimi, 2012). The reason for this variation is not clear. Several studies indicate that up to 30% of people with type 2 diabetes and a GFR <60ml/min/1.73m^2^ have normal albuminuria (Bash, Selvin, Steffes, Coresh, & Astor, 2008; H. Kramer & Molitch, 2005; H. J. Kramer, Nguyen, Curhan, & Hsu, 2003). Patients with normal albuminuria and renal impairment were found to be older, had lower prevalence of obesity, lower diastolic blood pressure, lower prevalence of smoking, macrovascular disease and ischemic heart disease and lower HBA1c (Coll-de-Tuero et al., 2012). A recent study found that among diabetic patients with renal impairment (estimated GFR less than 60/ml/min/1.73m^2^), 56.6% had normal albuminuria, 30.8% had microalbuminuria and 12.6% had macroalbuminuria (Penno et al., 2011). Table 1 shows the prevalence of normal albuminuria among kidney disease patients in similar studies (H. J. Kramer et al., 2003; Parving, Lewis, Ravid, Remuzzi, & Hunsicker, 2006; Penno et al., 2011; Retnakaran, Cull, Thorne, Adler, & Holman, 2006; Thomas et al., 2009; Yokoyama et al., 2009). This matter was put to the test when several cohort studies looked into the course of microalbuminuria in diabetic patients and serial measurements of albuminuria revealed that a number of microalbuminuric patients reverted to normal albuminuria, see Table 2 (Perkins et al., 2007; Perkins et al., 2003; Tabaei, Al-Kassab, Ilag, Zawacki, & Herman, 2001; Giorgino et al., 2004; Hovind et al., 2004; Perkins, Ficociello, Roshan, Warram, & Krolewski, 2010). Growing evidence show that GFR decline can occur in patients with normal albuminuria (Jerums, 2012). This could suggest that albuminuria may not be as sensitive as a marker for kidney function decline as previously thought. Further studies should be carried out in Malaysia to determine the role of albuminuria in detecting a decline in kidney function and whether the absence of albuminuria in CKD patients is related to the use of angiotensin converting enzyme inhibitors. This will help Clinicians to make an informed decision when screening patients for CKD.

**Table 1:** The prevalence of Normal Albuminuria in CKD Patients

1^st^ Author	Study Population	Patients with GFR < 60ml/min	Percentage of patients with GFR<60ml & normal albuminuria
(H. J. Kramer et al., 2003)	Type 2 diabetes	171	30%
(Parving et al., 2006)	Type 2 diabetes	2546	17%
(Retnakaran et al., 2006)	Type 2 diabetes	1132	51%
(Yokoyama et al., 2009)	Type 2 diabetes	506	51.7%
(Thomas et al., 2009)	Type 2 diabetes	920	55%
(Penno et al., 2011)	Type 2 diabetes	2959	56.6%

**Table 2:** The course of Microalbuminuria in diabetic patients

1^st^ Author	Study Population	Follow up period(years)	Patients with Microalbuminuria	Progressed to Macroalbuminuria	Regress to Normal
(Tabaei et al., 2001)	Diabetic patients	7	At start of follow up 23	6%	56%

(Perkins et al., 2003)	Type 1 diabetes	8	386	19	58

(Giorgino et al., 2004)	Type 1 diabetes	7	351	13.9%	50.6%

(Hovind et al., 2004)	Type 1 diabetes	7,5	79	34%	35%

(Perkins et al., 2007)	Type 1 diabetes	8-12	At start of follow up 301	31%	35.58%

(Perkins et al., 2010)	Type 1 diabetes	12	79	27	39

### 4.2 Serum Cystatin C

Cystatin C is a 13 kDa protein filtered by the glomeruli and reabsorbed and catabolised by epithelial cells of the proximal tubule with only small amounts excreted in the urine. Serum cystatin C has been proposed as a new marker for renal function and is under evaluation for GFR estimation. Earlier studies suggested that cystatin C is a better alternative than Creatinine because it is not affected by muscle mass (Baxmann et al., 2008; Mussap et al., 2002; Perlemoine et al., 2003) and that makes it a better marker than Creatinine in children, elderly and in patients with reduced muscle mass (Chew, Saleem, Florkowski, & George, 2008; Filler et al., 2005). More research on cystatin C found that factors other than renal function (that also affect serum creatinine) do affect serum cystatin C but to a lesser extent (Mussap et al., 2002; Pucci et al., 2007). Wei et al found that serum cystatin C is raised with age but as GFR start to decrease, the effect of age would reduce with the decreasing GFR which then becomes the most influencing factor on cystatin C (Wei, Ye, Pei, Wu, & Zhao, 2014). Two meta-analysis found cystatin C to be superior to creatinine as a marker for GFR (Dharnidharka, Kwon, & Stevens, 2002; Roos, Doust, Tett, & Kirkpatrick, 2007). Several studies found cystatin C to be more accurate than creatinine in detecting early diabetic nephropathy (Christensson et al., 2004; D. Willems, 2008; Hojs, Bevc, Ekart, Gorenjak, & Puklavec, 2006; Kim et al., 2013; Kimura et al., 2009; McNamara et al., 2009; Mussap et al., 2002; Perkins et al., 2005; Perlemoine et al., 2003; Rigalleau et al., 2008; Rule, Bergstralh, Slezak, Bergert, & Larson, 2006; Wei et al., 2014) See Table 3. Cystatin C can detect renal impairment in type 2 diabetic patients with normal albuminuria (Jeon et al., 2011). In addition, cystatin C has a role in identifying patients with CKD who are at higher risk for complication (Pavkov et al., 2013; Peralta et al., 2011) suggesting that cystatin C can predict patients at risk and warrant early referral. Very few studies looked into the role of cystatin C in detection of renal impairment in Malaysia (Marwyne et al., 2011; Zati Iwani AK, 2013). The high cost and limited availability of cystatin C test restricted its application in clinical practice. The introduction of certified reference material for cystatin C in 2010 intended to overcome the differences brought upon by using different calibrators and assays for cystatin C (Grubb et al., 2010). Recent guidelines from National Kidney Foundation recommend the use of cystatin C to validate the diagnosis of CKD in adults with GFR between 45-59 ml/min/1.73m^2^ and no other markers of kidney damage (Kidney disease: Improving global outcomes ckd work group. Clinical practice guideline for the evaluation and management of chronic kidney disease. [KDIGO], 2012). In light of this, future studies in Malaysia should evaluate the role of cystatin C in detecting early renal impairment and take into consideration the different ethnic groups when determining reference interval for cystatin C.

**Table 3:** Comparison between Cystatin C and Creatinine in predicting early renal impairment

1^st^ Author	population	Estimated GFR	Measured GFR	Performance
(Mussap et al., 2002)	52	Cockroft-Gault	^51^Cr-EDTA	Cystatin C superior to Creatinine

(Perlemoine et al., 2003)	89	Cockroft-Gault	^51^Cr-EDTA	Cystatin C superior to Creatinine

(Christensson et al., 2004)	123	NA	^51^Cr-EDTA	Cystatin C superior to Creatinine

(Perkins et al., 2005)	30	NA	Iothalamate clearance	Cystatin C superior to Creatinine

(Hojs et al., 2006)	164	Cockroft-Gault and MDRD	^51^Cr-EDTA	Cystatin C superior to Creatinine

(Rule et al., 2006)	460	Cockroft-Gault and MDRD	Iothalamate clearance	Cystatin C superior to Creatinine

(Rigalleau et al., 2008)	124	Cockroft-Gault and MDRD	^51^Cr-EDTA	Cystatin C superior to Creatinine

(D. Willems, 2008)	67	MDRD	^51^Cr-EDTA	Cystatin C superior to Creatinine

(McNamara et al., 2009)	48	Cockroft-Gault and MDRD	NA	Cystatin C superior to Creatinine

(Kimura et al., 2009)	289	Japanese modified MDRD	NA	Cystatin C superior to Creatinine

(Jeon et al., 2011)	332	MDRD and CKD-EPI	NA	Cystatin C superior to Creatinine

(Pavkov et al., 2013)	234	MDRD and CKD-EPI 2009, 2012 equation	Iothalamate clearance	Cystatin C superior to Creatinine

(Wei et al., 2014)	800		99mTc-DTPA	Cystatin C superior to Creatinine

### 4.3 Glomerular Filtration Rate (GFR)

The GFR is regarded as the best indicator for renal function. Accurate GFR estimation is important for the diagnosis and management of kidney disease (Stevens, Li, et al., 2011). The gold standard method for measuring GFR using an exogenous filtration markers (e.g. inulin, iohexol, ^99m^diethylenetriaminepentaacetic acid, ^125^I-iothalamate and ^51^Cr-ethylenediaminetetraacetic acid) is expensive, time consuming and inconvenient for patients. Several equations have been developed to estimate GFR and serum creatinine is used in those equations (Cockcroft & Gault, 1976; K/doqi clinical practice guidelines for chronic kidney disease: Evaluation, classification, and stratification, 2002; A. S. Levey et al., 1999) See Table 4. Nevertheless, serum creatinine has many limitations because it is affected by factors other than renal function, such as age, gender, race, muscle weight, diet and certain medications (Lamb, 2011; Mussap et al., 2002; Stevens, Coresh, Greene, & Levey, 2006). Moreover, kidney function can be reduced up to 50% and serum creatinine is still within normal range. Current guidelines from the National Kidney Foundation state that GFR should be estimated in all patients undergoing screening for CKD (Kdigo, 2012). The majority of GFR estimating equations were developed in the western countries, and despite the fact that Asia ranks high for prevalence of end stage renal disease, Asian countries do not have a unified GFR estimation equation.

**Table 4:** GFR Estimating Equations

Year	Equation	
1976	Cockroft-Gault(Cockcroft & Gault, 1976)	[(140-Age)×Weight](72×SCr)×0.85 (if female)

2002	4-variable MDRD(KDIGO 2002)	186×(SCr)^-1.154^×(Age)^-0.203^×(0.742)(if female)

2006	Re-expressed MDRD for standardized Creatinine (A. S. Levey et al., 2006)	175×(SCr)^-1.154^×(Age)^-0.203^×(0.742) (if female)×(1.212)(if black)

2006	Chinese modified MDRD(Ma et al., 2006)	(4-variable MDRD)×1.233

2007	Japanese modified MDRD(Imai et al., 2007)	(4-variable MDRD)×0.741

2009	Japanese modified MDRD(Matsuo et al., 2009)	(re-expressed MDRD)×0.808

2011	Thai modified MDRD(Praditpornsilpa et al., 2011)	(re-expressed MDRD)×1.129

2011	Thai eGFR formula (Praditpornsilpa et al., 2011)	375.5×(SCr)^-0.848^×Age ^-0.364^×0.712 (if female)

2009	CKD-EPI creatinine(Andrew S. Levey et al., 2009)	141×min(SCr/k,1)^α^×max(SCr/k,1)^-1.209^×0.993^age^[×1.018 if female]×[1.159 if black], where SCr is serum creatinine, k is 0.7 if female and 0.9 if male, α is -0.329 if female and -0.411 if male, min is the minimum of SCr/k or 1, and Max is the maximum of SCr/k or 1.

2012	CKD-EPI cystatin C(Inker et al., 2012)	133×min(Scys/0.8,1)^-0.499^×max(Scys/0.8,1)^-1.328^×0.996^age^[×0.932 if female], Scys is Serum Cystatin C, min indicates the minimum of Scr/k or 1, and max indicates the maximum of Scys/k or1.

2012	CKD-EPI creatinine-cystatin C (Inker et al., 2012)	135×min(SCr/k,1)^α^×max(SCr/k,1)^-0.601^×min(Scys/0.8,1)^-0.375^×max max(Scys/0.8,1)^-0.711^×0.995^age^[×0.969 if female] ×1.08 if black, where SCr is serum creatinine, SCys is serum cystatin C, k is 0.7 if female and 0.9 if male, α is -0.248 if female and -0.207 if male, min indicate the minimum of SCr/k or 1, and max indicate the maximum of SCr/k or 1.

#### 4.3.1 GFR Estimation by the MDRD Equation

The MDRD equation was introduced in 1999 and is used to estimate GFR. The equation was developed in 1628 Caucasians and African Americans patients with impaired kidney function (A. S. Levey et al., 1999). The equation has been re-expressed in 2006 for standard creatinine to improve its accuracy (A. S. Levey et al., 2006). The standardization of serum creatinine came as a big step to overcome the variability caused by using different measures for serum creatinine. The MDRD equation is used in Asia to estimate GFR, but being dependant on creatinine does not make it an ideal equation. Creatinine is influenced by muscle mass and Asian are different from Caucasian and from each other in their body mass index/body fat % relationship (Deurenberg, Deurenberg-Yap, & Guricci, 2002; Wen, Rush, & Plank, 2010). In China the MDRD equation was modified with a racial coefficient (Ma et al., 2006), also in Japan (Imai et al., 2007; Matsuo et al., 2009) and in Thailand (Praditpornsilpa et al., 2011). See Table (3). Adding a racial coefficient led to better GFR estimation but there was a difference between the Chinese and Japanese coefficient. The variation could be attributed to methodological rather than ethnic difference because the reference GFR measurement and creatinine measurements were different in both studies (Ho & Teo, 2010; Rule & Teo, 2009). In Malaysia, the MDRD equation was adopted without modification.

#### 4.3.2 GFR estimation by The CKD-EPI Creatinine Equation

The Chronic Kidney Disease Epidemiology Collaboration (CKD-EPI) group introduced a new equation in 2009. The CKD-EPI creatinine equation was developed in 8254 patients and validated in 3896 patients including patients with and without kidney disease (A. S. Levey et al., 2009). The CKD-EPI equation retained the precision and accuracy of the MDRD equation at GFR < 60 ml/min/1.73m^2^, showed less bias and improved precision at GFR>60ml/min/1.73m^2^ (Stevens, Claybon, et al., 2011). The MDRD equation was known for underestimating GFR higher than 60ml/min/1.73m^2^ (de Boer & Steffes, 2007; Rigalleau et al., 2011). The CKD-EPI equation is reportedly more accurate and resulted in lower prevalence of estimated GFR< 60 ml/min/1.73m^2^. Patients who were reclassified to higher GFR using the CKD-EPI equation had lower risk of death and less likely to have CKD risk factors or co-morbid conditions based on data extracted from longitudinal studies (Kitiyakara et al., 2012; Matsushita et al., 2012; Shafi et al., 2012; Stevens, Li, et al., 2011; Targher et al., 2012; White, Polkinghorne, Atkins, & Chadban, 2010) See Table 5. Although one study reported that the MDRD equation was more appropriate for risk stratification in 85 year old patients (Willems et al., 2013). The CKD-EPI equation was validated in different population and was found to have lower bias and more accuracy compared to the MDRD equation (Du et al., 2011; Jeong et al., 2013; Jessani et al., 2014; Stevens et al., 2010; Teo et al., 2011). Currently the reporting limit for estimating GFR with the MDRD equation is set at 60ml/min/1.73m^2^ and GFR in the range of 60-90ml/min/1.73m^2^ (although being CKD stage 1-2) falls under GFR >60ml/min/1.73m^2^ (de Boer & Steffes, 2007). But with CKD-EPI equation the reporting limit has been increased to 90ml/min/1.73m^2^ (Johnson et al., 2012). In Asian countries, namely China, Japan and Korea, adding an ethnic coefficient to the CKD-EPI equation led to better performance (Horio, Imai, Yasuda, Watanabe, & Matsuo, 2010) (Du et al., 2011; Jeong et al., 2013). In Singapore, a multiracial country similar to Malaysia, the CKD-EPI creatinine equation did not need an ethnic coefficient because the derived coefficients were very close to each other (Teo et al., 2011). In Malaysia, the CKD-EPI equation is yet to be validated. Population based studies should evaluate the performance of the new equation and take into account the aborigines and whether an ethnic coefficient is necessary.

**Table 5:** Comparison between the MDRD And CKD-EPI Equation For Risk Stratification And CKD Prevalence

1^st^ Author	Study Population	Years of follow up	Performance of CKD-EPI equation to estimate GFR	Percentage of CKD patients Reclassified using CKD-EPI	Prevalence of CKD using CKD-EPI equation (compare to MDRD)
(White et al., 2010)	11,247	7.5	CKD-EPI improve risk stratification	4.54% of CKD grade 3a to CKD	13.4% to 11.5%

(Stevens, Li, et al., 2011)	116,321	3.7	CKD-EPI improve risk stratification	24.4% of CKD grade 3a to higher GFR	16.8% to 14.3%

(Shafi et al., 2012)	16,010	18	CKD-EPI improve risk stratification	19.4% of CKD grade 3 to higher GFR	45.6% to 28.8%

(Targher et al., 2012)	2,823 type 2 diabetic	6	CKD-EPI improve risk stratification for all cause mortality and CVD	NA	22% to 20.2

(Matsushita et al., 2012)	13,905 middle age without history of CVD	16.9	CKD-EPI improve risk stratification	44.9% of CKD grade1&2 and 43.5% of CKD grade 3 to higher GFR	From 2.5% to 1.4%

#### 4.3.3 GFR Estimation by Cystatin C Equation

Cystatin C has been proposed as a new marker to detect early renal function decline in the absence of albuminuria, predict renal impairment when GFR is still normal or elevated and also has a role as risk predictor to warrant early referral. Serum cystatin C is also affected by non-renal factors, though to a lesser extent compared to serum creatinine. Several cystatin C based equations have been developed to estimate GFR. Some of those equations incorporated both cystatin C and creatinine and they performed fairly well (Ma et al., 2007). Cystatin C based equations improved GFR estimates in CKD patients (Krolewski et al., 2012; Lopes et al., 2013; Rigalleau et al., 2008; Rule et al., 2006). In 2012 The CKD-EPI group introduced GFR estimating equation that incorporates cystatin C and one that incorporates both cystatin C and creatinine. The two equations were developed in 5352 participant from 13 studies and the combination equation (that includes cystatin C and creatinine) performed better than equations based on either of them alone, suggesting that errors due to non renal determinants of creatinine and cystatin C are smaller in equation that combines both of them. The researchers recommend that cystatin C should not replace creatinine, instead, an equation combining both of them provides more accurate GFR estimates for certain groups (Inker et al., 2012). Another comparison between the new CKD-EPI equation based on cystatin C alone and a combination of cystatin C and creatinine in kidney transplant recipient also found that both equation performed better than the CKD-EPI creatinine equation (Masson et al., 2013). In a meta-analysis of 11 general population studies (including a total of 90,750 participants) and 5 cohort on CKD patients (including a total of 2960 participants) found that estimating GFR using the CKD-EPI cystatin C equation and the CKD-EPI cystatin C and creatinine equation strengthened the association between GFR categories and the risk of death and end stage renal failure across diverse populations. The study also found that 42% of participants with GFR 45-59ml/min/1.73m^2^ using the creatinine based GFR where reclassified to GFR > 60ml/min/1.73m^2^ using the cystatin C equation. Patients who were reclassified to higher GFR had 34% reduction in risk of death and 80% reduction in risk of end stage renal disease (Shlipak et al., 2013). In Singapore, the CKD EPI equation 2012 (using both cystatin C and creatinine) were evaluated and found that a combination of both markers increase precision without having to add an ethnic coefficients (Teo et al., 2012). Currently, the National Kidney Foundation recommends the use of cystatin C based equation to estimate GFR whenever cystatin C is ordered (Kdigo, 2012).

## 5. Conclusion and Recommendation

Screening for CKD in Malaysia is based on creatinine and albuminuria. Creatinine is known for its limitation, being affected by non-renal factors and rising only after substantial kidney damage has occurred. The GFR should be reported by laboratories whenever serum creatinine is ordered, so as not to miss patients who have a declining GFR and a seemingly normal creatinine. The MDRD equation is currently used (without ethnic coefficient) to estimate GFR, but being dependant on creatinine puts at a disadvantage. The new CKD-EPI equation is a promising new measure for GFR estimation having performed well compared to the MDRD equation. This needs validation in multiethnic Malaysian population. Population based study should be undertaken to determine whether an ethnic coefficient is needed for the new equation.

Albuminuria may not be as sensitive as a marker as previously thought. Recent studies show that GFR decline can occur in the absence of albuminuria. Research should be undertaken in Malaysia to further evaluate the role of albuminuria in detecting early decline in GFR. Cystatin C was proposed as a new marker for renal impairment. Cystatin C is shown to be superior to creatinine in detecting early renal function decline. This needs further evaluation in Malaysia to determine the exact role of cystatin C as compared to other CKD markers. Reference interval for cystatin C should be established for Chinese, Indians and Malays in Malaysia.
